# THE EFFECTS OF THE NAMASTE CARE FAMILY PROGRAM ON QUALITY OF LIFE OF PEOPLE WITH ADVANCED DEMENTIA

**DOI:** 10.1093/geroni/igz038.3036

**Published:** 2019-11-08

**Authors:** Jenny T van der Steen, Karlijn J Joling, Anneke L Francke, Wilco P Achterberg, Hanneke J A Smaling

**Affiliations:** 1 Leiden University Medical Center, Leiden, Netherlands; 2 Amsterdam University Medical Centers, Amsterdam, Netherlands

## Abstract

People with advanced dementia often die in nursing homes. Family caregivers frequently feel that their loved one’s quality of life and dying is suboptimal. The daily Namaste Care Family program -derived from the US Namaste Care program- involves family caregivers and integrates personalized care with meaningful activities for people with advanced dementia. A cluster-randomized controlled trial (December 2016 - December 2018) examined effects of the Namaste Care Family program on resident and family caregiver outcomes. Ten Dutch nursing homes implemented Namaste Care Family for 117 residents, while nine nursing homes provided usual care for 114 residents in the study. Nursing staff assessed quality of life over the last week with the Quality of Life in Late-Stage Dementia (QUALID, the primary resident outcome measure). Research assistants observed discomfort during the sessions with the Discomfort Scale-Dementia of Alzheimer Type (DS-DAT). Assessments were at baseline and after 1, 3, 6, and 12 months. We found a significant difference in QUALID score at 12 months favoring quality of life in the intervention group. Further, the intervention group showed less signs of discomfort at 3, 6, and 12 months compared with the control group. The Namaste Care Family program can improve quality of life of people with advanced dementia in the long run. These study findings support sustained implementation of the daily program in nursing homes. Further analyses of effects on the other outcomes will include blinded DS-DAT assessments, more secondary outcome measures and family caregiver outcomes.

